# Foreign-born status and risk of gestational diabetes mellitus by years of residence in the United States

**DOI:** 10.1038/s41598-023-36789-8

**Published:** 2023-06-21

**Authors:** Akaninyene I. Noah, Maria J. Perez-Patron, Megha Gongalla, Ashley V. Hill, Brandie DePaoli Taylor

**Affiliations:** 1 Division of Basic Science and Translational Research, Department of Obstetrics and Gynecology, University of Texas Medical Branch, MRB, 11.158A, 301 University Blvd, Galveston, TX 77555 USA; 2 Department of Epidemiology and Biostatistics, School of Public Health, Texas A&M University, College Station, TX USA; 3 Department of Sociology, College of Liberal Arts, Temple University, Philadelphia, PA USA; 4 Department of Epidemiology, Graduate School of Public Health, University of Pittsburgh, Pittsburgh, USA; 5 Department of Population Health and Health Disparities, University of Texas Medical Branch, Galveston, TX USA

**Keywords:** Metabolic disorders, Risk factors

## Abstract

To explore the association between acculturation among foreign-born (FB) women, gestational diabetes (GDM) and GDM-associated adverse birth outcomes, we conducted a retrospective cohort study of 34,696 singleton pregnancies from Houston, TX, between 2011 and 2022. FB women (n = 18,472) were categorized based on years of residence in US (0–5, 6–10, and > 10 years), while US-born women (n = 16,224) were the reference group. A modified Poisson regression model determined the association between acculturative level and GDM within the entire cohort and stratified by race/ethnicity. Compared to US-born women, FB women with 0–5 years [adjusted relative risk (RRadj.) 1.27, 95% confidence interval [CI] 1.14–1.42)], 6–10 years (RRadj. 1.89, 95%CI 1.68–2.11) and > 10 years in the US (RRadj. 1.85, 95%CI 1.69–2.03) had higher risk of GDM. Results were consistent for all racial/ethnic groups, although associations were not significant at 0–5 years. FB women had lower risk of other adverse pregnancy outcomes, except for preeclampsia with severe features at higher levels of acculturation. Results were similar among those with and without GDM. In conclusion, FB status increases risk of GDM among all racial/ethnic groups but is elevated with higher acculturation levels.

## Introduction

Gestational diabetes mellitus (GDM) is defined as the first onset of insulin intolerance during pregnancy and is one of the most frequently occurring metabolic disorders of pregnancy^1^. Recent estimates place the annual prevalence of GDM in the United States at approximately 7.6%^2^. GDM is associated with increased risk of adverse birth outcomes such as preeclampsia, Cesarean section, macrosomia, and preterm delivery^3^. In addition, those diagnosed with GDM have an increased risk of developing Type 2 diabetes within two years postpartum, and infants born to GDM pregnancies have an elevated risk of metabolic disorders later in life^4^. Thus, GDM is a significant public health concern, and there is a need to understand population-level factors that increase the risk of GDM and GDM-associated complications.

Foreign-born immigrants have higher rates of GDM than their native-born counterparts, despite typically experiencing better health outcomes in a phenomenon commonly called the healthy migrant effect^5^. This health advantage appears to wane with increased acculturation, which can be defined as gradual socio-cultural assimilation to the dominant culture^6^. A recent study among foreign-born Asian women showed a negative relationship between increased acculturation and GDM risk^7^. Although GDM was not examined, other studies among Hispanic, African and White immigrants show an increase in diabetes risk^8–10^. The relationship between acculturation and GDM is unclear across different race/ethnic groups.

Our primary objective was to examine the association between foreign-born status (FBS) and GDM across different race/ethnicity groups. In addition, we investigated the effect of acculturation, using years in the US as our proxy, on GDM risk among foreign-born women. As a secondary analysis, we examined the association between different acculturation levels in foreign-born women on GDM-associated adverse birth outcomes.

## Methods

### Study design and population

We conducted a retrospective cohort study of singleton pregnancies in the Peribank database^11^. Briefly, Peribank was developed by Baylor College of Medicine and recruits women from four hospitals in Houston, Texas, at the time of admission to labor and delivery. Demographic and clinical variables were obtained through interviews and electronic health records. A board-certified obstetrician performs regular audits of the data to maintain high reliability. All participants provided informed consent. The Governance Board of Peribank provided approval for the current study. The University of Texas Medical Branch Institutional Review Board reviewed this study and determined it to be exempt.

### Study population and exclusion criteria

We identified 49,841 singleton pregnancies between August 2011 and April 2022. Only the first recorded delivery was used in cases where multiple births occurred within that timeframe, and any subsequent birth was excluded (n = 6044). Patients with missing self-reported country of birth (n = 1593) and time spent in the US (n = 7508) were also excluded from our study. After all exclusions, we had a final analytic sample of 34,696 singleton deliveries.

### Foreign-born status (FBS) and years lived in the US

Patients were categorized by race and ethnicity as non-Hispanic White (White), non-Hispanic Black (Black), non-Hispanic Asian (Asian), and Hispanic. Patients’ self-reported country of birth was used to determine foreign-born status (FBS). Women who listed ‘USA’ as their country of birth were indicated as US born, while those with a non-US country were listed as foreign-born. Self-reported time spent in the US by foreign-born women was used as our proxy for acculturation. We used 5-year increments, i.e., 0–5, 6–10, and greater than 10 years (> 10), as our indicator for the level of acculturation. Prior research shows that after 5 years or more in the US, foreign-born women begin to show a decline in the immigrant health advantage^12^.

### Primary outcome

Gestational diabetes mellitus (GDM) was our primary outcome of interest. Diagnoses of GDM followed standardized hospital protocols typically using a two-step screening process between 24 and 28 gestation weeks. The first step was administering a 50 g oral glucose test with a 140 mg/dl cutoff indicator, while the second was a 100 g oral glucose tolerance test^13^. Only 118 women (0.3%) were not screened for GDM.

### Secondary outcomes

For our secondary analysis, we examined the association between acculturation level and GDM-associated adverse birth outcomes. The outcomes examined included: Cesarean section delivery (C-section), gestational hypertension, superimposed preeclampsia, preeclampsia, preeclampsia with severe features, medically indicated preterm birth (PTB) and spontaneous PTB. Preeclampsia was diagnosed based on ACOG criteria, as the new onset of hypertension, along with proteinuria > 300 mg in a urine sample or in the absence of proteinuria evidence of multisystem failure^14^. Preeclampsia with severe features was defined as preeclampsia with one or more of the following signs: (1) multi-organ failure, (2) systolic blood pressure > 160 mmHg or diastolic blood pressure > 110 mmHg, (3) proteinuria, (4) reduced platelet count (5) intrauterine growth restriction^14^.

Superimposed preeclampsia was defined among those with chronic hypertension who went on to develop preeclampsia symptoms^14^. Gestational hypertension was defined as the presence of newly occurring hypertension after 20 gestation weeks without any other preeclampsia symptoms^14^. PTB was defined as a live birth occurring before 37 gestation weeks, where gestational age was determined using ACOG criteria using last menstrual period and correction by ultrasound when needed^15^. Medically indicated PTB was defined as defined as a preterm birth, medically induced due to maternal or fetal distress, while spontaneous PTB occurred due to preterm labor or rupture of membranes^16^.

### Covariates

Demographic variables obtained from Peribank included maternal age, education (> than high school, high school degree or less), insurance method (private, Medicaid/Children’s health insurance program (CHIP), no insurance/other), smoking, and alcohol use. We also obtained data on maternal health history and current health indicators such as pre-pregnancy body mass index (BMI), prior GDM, mental health issues, chronic health conditions (cardiovascular disease, asthma, chronic hypertension), and gravidity. Mental health issues were defined as the presence of either anxiety or depression.

### Statistical analysis

We compared maternal demographics and clinical variables between foreign-born and US-born women, stratified by race/ethnicity, using the Chi-squared test (Fisher’s exact test if cell size < 5) and T-test to determine the presence of significant differences between groups. Next, we examined the distribution of years in US among foreign-born women, stratified by race/ethnicity, both as a categorical (0–5, 6–10 and > 10) and continuous variable. The distribution of years in US, among the most frequently reported countries within each race/ethnic group was also examined.

To determine the association between FBS and GDM, we used modified Poisson regression models^17^ to calculate the relative risk (RR) and 95% confidence intervals (CI), a method frequently used to measure relative risk^18^. A log-binomial model was used initially but there were converge issues when adding covariates to our model. To select our confounders, we used a directed acyclic graph (DAG)^19^. However, acknowledging that we do not fully know the relationship between years in the US and some variables such as development of chronic health conditions, we used the disjunctive cause criterion, which includes covariates that are causes of exposure, causes of outcome, or both^20^. Considering criterion and the DAG we created^21^ (Supplementary Fig. S5), we decided to utilize 3 models for our analysis. Model 1 was unadjusted, model 2 adjusted for maternal age only, and model 3, our fully adjusted model, adjusted for maternal age, pre-pregnancy BMI, insurance method, and chronic health conditions. We considered prior GDM, however this variable is most likely a mediator for some pregnancies. As a sensitivity analysis we conducted a mediation analysis, using VanderWeele’s causal mediation macro^22^, to determine if prior GDM has a mediating effect between FBS and GDM in the current pregnancy. We found some evidence of mediation, therefore, prior GDM was not adjusted for in the model.

We stratified our foreign-born population by time spent in the US (0–5, 6–10, > 10 years), using US-born women as our reference group. We explored this analysis among the entire cohort and stratified by race/ethnicity, utilizing the same 3 models as our prior analysis. As a sensitivity analysis we evaluated the association between years in US, as a continuous variable, and GDM among foreign-born women, stratified by race/ethnicity using a spline regression model^23^. Lastly, we examined the association between acculturation and adverse birth outcomes using the methods described above. As GDM is known to increase risk of several adverse birth outcomes, we stratified these analyses by GDM status^24^.

The missingness among the variables in our models ranged from 0.3 to 8.9%. To account for missing data, we used a fully conditional specified multiple imputation with 10 iterations. As a sensitivity analysis we also conduced our primary analysis, looking at the association between FBS and GDM, using complete case analysis. However, effect estimates were nearly identical [e.g., for association between FBS and GDM: (RRadj. 1.62, 95% CI 1.49–1.77) vs. using multiple imputation (RRadj. 1.64, 95% CI 1.54–1.83)], therefore results are shown using the multiple imputation model. All analysis was conducted using SAS version 9.4, Cary, North Carolina.

## Results

### Study demographics

Our study population consisted of 34,696 women, of which 16,224 (46.8%) were US-born, and 18,472 (53.3%) were foreign-born (Table 1). Among our study population, 20,382 women (58.7%) self-identified as Hispanic, 7089 (20.4%) as White, 5097 (14.7%) as Black, 1818 (5.2%) as Asian, and 301 (0.9%) as Other. Hispanic and Asian foreign-born women were more likely to have a high school or less education (Hispanic; 83.4% vs. 47.4%; Asian; 13.2% vs. 2.0%) compared to their US-born counterparts. While among Black women, US-born women were more likely to have a high school or less education (37.3% vs. 30.7%). Across all race/ethnic groups, US-born women were more likely to use alcohol during pregnancy (Black; 69.2% vs. 19.4%; Hispanic; 65.8% vs. 23.4%; Asian 78.8% vs. 44.8%; White; 84.0% vs. 66.2%) relative to their foreign-born counterparts. We also observed this trend in smoking during pregnancy, except for White women, where the difference was not significant. Foreign-born women across all race/ethnic groups were also less likely to have a chronic health condition (Black; 10.4% vs. 24.5%; Hispanic; 7.5% vs. 14.3%, Asian; 8.1% vs. 13.5%; White; 12.1% vs. 19.7%). However, they were more likely to have prior GDM (Black; 3.3% vs. 1.2%; Hispanic; 5.8% vs. 2.0%; Asian 3.1% vs. 1.4%) compared to their US-born counterparts, among White women the difference was not significant.

**Table 1:** Distribution of maternal demographic, social and health variables by foreign-born (FB) status, stratified by race and ethnicity.

Characteristics	Non-Hispanic Black (n,% = 5097, 14.7)			Hispanic (n, % = 20,382, 58.7)			Non-Hispanic Asian (n, % = 1818, 5.2)			Non-Hispanic White (n, % = 7089, 20.4)		
	US-born (n = 3842)	FB (n = 1255)	*P	US-born (n = 5415)	FB (n = 14,967)	*P	US-born (n = 511)	FB (n = 1307)	*P	US-born (n = 6363)	FB (n = 726)	*P
Maternal demographic characteristics												
Maternal age												
Median(IQR)	27 (23–32)	32(29–36)	< 0.001	26(22–30)	30 (25–34)	< 0.001	33 (31–35)	32 (29–36)	0.03	31 (28–34)	32 (29–36)	< 0.001
Marital status												
Married	1978 (51.7)	961 (77.4)	< 0.001	3805 (70.8)	10,027 (67.4)	< 0.001	493 (96.7)	1248 (96.2)	0.60	5807 (91.6)	677 (93.4)	0.10
Single	1845 (48.3)	281 (22.6)		1572 (29.2)	4845 (32.6)		17 (3.3)	50 (3.9)		532(8.4)	48 (6.6)	
Education												
> High school	2369 (62.7)	844 (69.4)	< 0.001	2813 (52.6)	2413 (16.6)	< 0.001	498 (98.0)	1109 (86.8)	< 0.001	5653 (89.8)	620 (87.6)	0.07
High school or less	1409 (37.3)	373 (30.7)		2538 (47.4)	12,136 (83.4)		10 (2.0)	169 (13.2)		642 (10.2)	88 (12.4)	
Insurance												
Private	1070 (28.4)	210 (17.0)	< 0.001	1557 (29.2)	1000 (6.7)	< 0.001	469 (93.6)	892 (69.5)	< 0.001	5119 (82.5)	520 (72.8)	< 0.001
Medicaid/CHIP	2612 (69.3)	908 (73.4)		3661 (68.7)	12,643 (85.0)		32 (6.4)	353 (27.5)		1031 (16.6)	168 (14.0)	
Unknown/no insurance	90 (2.4)	119 (9.6)		110 (2.1)	1234 (8.3)		0 (0)	37 (2.9)		57(0.9)	26(3.6)	
Social and behavioral characteristics												
Alcohol use												
No	1182 (30.8)	1010 (80.6)	< 0.001	1851(34.2)	11,455 (76.7)	< 0.001	108 (21.2)	720(55.2)	< 0.001	1017 (16.0)	245 (33.8)	< 0.001
Yes	2656 (69.2)	243 (19.4)		3562 (65.8)	3490 (23.4)		402 (78.8)	584 (44.8)		5349 (84.0)	479 (66.2)	
Smoking												
No	3077 (80.1)	1225 (97.8)	< 0.001	4520 (83.5)	14,102 (94.4)	< 0.001	449 (88.0)	1224 (93.9)	< 0.001	4929 (77.6)	558 (77.1)	0.77
Yes	764 (19.9)	28 (2.20)		891 (16.5)	842 (5.6)		61 (12.0)	80 (6.1)		1427 (22.5)	166 (22.9)	
Clinical and health history characteristics												
Pre-pregnancy body mass index												
Median (IQR)	27.3 (23.2–32.8)	26.6 (23.2–30.5)	< 0.001	27.5 (23.4–32.6)	26.6 (23.4–30.7)	< 0.001	22.3 (20.3–25.5)	22.3 (20.3–25.5)	0.20	23.8 (21.3–28.1)	22.8 (20.7–26.3)	< 0.001
Chronic health condition												
No	2895 (75.5)	1117 (89.6)	< 0.001	4628 (85.7)	13,753 (92.5)	< 0.001	441 (86.5)	1119 (92.0)	< 0.001	5095 (80.3)	637 (87.9)	< 0.001
Yes	940 (24.5)	130 (10.4)		772 (14.3)	1119 (7.5)		69 (13.5)	105 (8.1)		1247 (19.7)	88 (12.1)	
Prior GDM												
No	3790 (98.8)	1206 (96.7)	< 0.001	5294 (98.0)	14,016 (94.2)	< 0.001	503 (98.6)	1263 (96.9)	0.04	6270 (98.9)	711 (98.1)	0.06
Yes	45 (1.2)	41 (3.3)		106 (2.0)	856 (5.8)		7 (1.4)	41 (3.1)		72 (1.1)	14 (1.9)	
Mental health issues												
No	3197 (82.4)	1213 (97.3)	< 0.001	4633 (85.8)	14,227 (95.7)	< 0.001	464 (91.0)	1225 (93.9)	0.03	4667 (73.6)	634 (87.5)	< 0.001
Yes	638 (94.9)	34 (2.7)		767 (14.2)	645 (4.3)		46 (9.0)	79 (6.1)		1675 (26.4)	91 (12.6)	
Gravidity												
No prior pregnancy	1224 (31.9)	295 (23.5)	< 0.001	1899 (35.1)	2961 (19.8)	< 0.001	230 (45.1)	485 (37.2)	0.01	2452 (38.6)	340 (46.9)	0.75
1–2 prior pregnancies	1687 (43.9)	588 (46.9)		2410 (44.5)	6900 (74.1)		232 (45.5)	668 (51.2)		2913 (45.8)	340 (10.6)	
> 2 prior pregnancies	930 (24.2)	371 (29.6)		1102 (20.4)	5084 (34.0)		48 (9.4)	152 (11.7)		992 (15.6)	106 (10.2)	

IQR; interquartile range, CHIP; children’s health insurance program, GDM; gestational diabetes.

*P-value calculated by T-test, Chi-squared test or fishers exact test as appropriate.

### Distribution of years in US among foreign-born women

We explored the distribution of years in the US among foreign-born women. Among our foreign-born study population, 7610 women (41.2%) had spent > 10 years in the US, followed by 6947 (37.6%) and 3915 (21.2%) women in the 0–5 and 6–10-year ranges respectively (Table 2). Among foreign-born Black women, the distribution skewed towards those with less time in the US; those in the 0–5 (61.7%) and 6–10 (22.2%) year groups accounted for over 80% of the population. The majority of foreign-born Black women were from Nigeria, accounting for about 50% of the Black immigrant population.

**Table 2:** Distribution of years in US among foreign-born (FB) women by country of birth, stratified by race and ethnicity.

	Overall, N (%)	Stratified in 5-year increments			Distribution of years in US	
		0–5 Years	6–10 Years	> 10 Years	Mean (Stdev.)	Min, Max
All FB women	18,498	6947 (37.6)	3915 (21.2)	7610 (41.2)	10.5 (9.0)	0, 78
Non-Hispanic Black FB women, overall and by country of birth						
Overall	1255	774 (61.7)	278 (22.2)	203 (16.2)	7.3 (9.4)	0, 77
Nigeria	635 (50.6)	456 (71.8)	83 (13.1)	96 (15.1)	5.7 (8.6)	0, 77
Ethiopia	80 (6.4)	43 (53.8)	21 (26.3)	16 (20.0)	7.7 (7.2)	0, 34
Cameroon	59 (4.7)	42 (71.2)	9 (15.3)	8 (13.6)	6.1 (10.8)	0, 77
Other	481 (33.3)	233 (48.4)	90 (18.7)	158 (32.9)	9.5 (10.0)	0, 77
Hispanic FB women, overall and by country of birth						
Overall	14,967	5321 (35.6)	3303 (22.1)	6343 (42.4)	10.4 (8.4)	0, 78
Mexico	7463 (49.9)	1505 (20.2)	1651 (22.1)	4307 (57.7)	12.9 (8.1)	0, 77
Honduras	2693 (18.0)	1497 (55.6)	568 (21.1)	628 (23.3)	6.9 (7.3)	0, 78
El Salvador	2474 (16.5)	1149 (46.5)	562 (22.7)	761 (30.8)	8.4 (7.4)	0, 77
Other	2339 (16.6)	1170 (50.0)	522 (22.3)	647 (27.7)	8.4 (8.6)	0, 77
Non-Hispanic Asian FB women, overall and by country of birth						
Overall	1307	457 (35.0)	248 (19.0)	602 (46.1)	13.3 (11.4)	0, 77
India	271 (20.7)	90 (33.2)	69 (25.5)	112 (41.3)	12.1 (10.6)	0, 43
China	178 (13.6)	58 (32.6)	51 (28.7)	69 (38.8)	11.9 (9.8)	0, 36
Vietnam	162 (12.4)	50 (30.9)	27 (16.7)	85 (52.5)	14.3 (10.9)	0, 40
Other	696 (53.3)	259 (37.2)	101 (15.2)	336 (48.3)	13.9 (12.1)	0, 77
Non-Hispanic White FB women, overall and by country of birth						
Overall	726	276 (38.0)	124 (17.1)	326 (44.9)	13.5 (12.0)	0, 77
United Kingdom	63 (8.7)	25 (39.7)	8 (12.7)	30 (47.6)	14.1 (12.4)	0, 37
Canada	50 (6.9)	19 (38.0 )	5 (10.0)	26 (52.0)	16.3 (12.9)	1, 38
Germany	47 (6.5)	10 (21.3)	5 (10.6)	32 (68.1)	20.9 (12.9)	0, 42
Other	566 (88.0)	222 (39.2)	106 (18.7)	238 (42.1)	12.5 (11.5)	0, 77

Among Hispanic, Asian, and White foreign-born women, the distribution of years in the US followed a similar pattern where most women had lived > 10 years in US (42.4%, 46.1%, and 44.9%, respectively), followed by more recent immigrants with 0–5 years in US (35.6%, 35.0%, and 38.0%, respectively) and those with 6–10 years in US (22.1%, 19.0%, and 17.1%, respectively).

Within the Hispanic foreign-born population, three countries, Mexico (49.9%), Honduras (18.0%), and El Salvador (16.5%), accounted for over 84% of the Hispanic population. In contrast there were numerous countries of birth for Asian and White women. Foreign-born Asian women from India (20.7%) followed by China (13.6%) and Vietnam (12.4%) were the most frequently reported in our Asian population. Foreign-born White women from the United Kingdom (8.7%), Canada (6.9%) and Germany (6.5%) were the most frequently reported in our White population.

### Association between FBS and GDM

We explored the association between FBS and GDM across the entire cohort and stratified by race/ethnicity. Among the entire cohort, FBS was associated with an increased risk of GDM (RRadj. 1.64, 95% CI 1.54–1.83) relative to US-born women (Table 3). Compared to US-born Black women, foreign-born Black women had an elevated risk of GDM (RRadj. 1.35, 95% CI 1.04–1.77). Foreign-born Hispanic (RRadj. 1.32, 95% CI 1.18–1.49), Asian (RRadj. 1.46, 95% CI 1.04–2.05), and White (RRadj. 1.41, 95% CI 1.05–1.89) women all had elevated GDM risk relative compared to their respective US-born counterparts.

**Table 3:** Association between foreign-born status and gestational diabetes (GDM), stratified by race and ethnicity.

	GDM− N, (%)	GDM+ N (%)	^a^Model 1 RR, (95% CI)	^b^Model 2 RR, (95% CI)	^c^Model 3 RR, (95% CI)
Entire Cohort					
US-born	15,160 (93.7)	1019 (6.3)	Ref	Ref	Ref
Foreign-born	16,094 (87.5)	2310 (12.6)	1.99 (1.85–2.15)	1.81 (1.68–1.94)	1.64 (1.54–1.83)
Non-Hispanic Black women					
US-born	3624 (94.7)	204 (5.3)	Ref	Ref	Ref
Foreign-born	1147 (91.8)	103 (8.2)	1.55 (1.22–1.96)	1.15 (0.90–1.47)	1.35 (1.04–1.77)
Hispanic women					
US-born	4989 (92.3)	416 (7.7)	Ref	Ref	Ref
Foreign-born	12,966 (86.9)	1948 (13.1)	1.70 (1.53–1.89)	1.34 (1.20–1.49)	1.32 (1.18–1.49)
Non-Hispanic Asian women					
US-born	465 (91.2)	45 (8.8)	Ref	Ref	Ref
Foreign-born	1119 (85.9)	184(14.1)	1.61 (1.16–2.23)	1.63 (1.18–2.25)	1.46 (1.04–2.05)
Non-Hispanic White women					
US-born	5995 (94.5)	349 (5.5)	Ref	Ref	Ref
Foreign-born	669 (92.7)	53 (7.3)	1.34 (1.00–1.79)	1.23 (0.92–1.65)	1.41 (1.05–1.89)

^a^Model 1 was unadjusted/crude.

^b^Model 2 adjusted for maternal age.

^c^Model 3 adjusted for maternal age, chronic health conditions, insurance type, and pre-pregnancy body mass index.

### Association between FBS and GDM, stratified by years in the US

Among our entire cohort, we observed foreign-born women with 0–5 years in the US had an increased risk of GDM (RRadj. 1.27, 95% CI 1.14–1.42); however, GDM risk was higher among those with 6–10 years (RRadj. 1.89, 95% CI 1.68–2.11) and > 10 years (RRadj. 1.85, 95% CI 1.69–2.03), compared to US-born women (Table 4). We observed a similar trend after stratifying by race/ethnicity. Compared to US-born Hispanic women, foreign-born Hispanic women with 0–5 years in the US (RRadj. 1.01, 95% CI 0.87–1.16) showed no association with GDM, but those with 6–10 years (RRadj. 1.46, 95% CI 1.27–1.69) and > 10 years in the US (RRadj. 1.45, 95% CI 1.28–1.64) had an elevated risk of GDM (Table 4). Foreign-born Black women followed a similar pattern where women in the 0–5 year range showed no significant association with GDM (RRadj. 1.10, 95% CI 0.79–1.55), whereas those with 6–10 years (RRadj. 1.59, 95% CI 0.97–2.53) showed a borderline association with GDM and those with > 10 years had higher GDM risk (RRadj. 1.75, 95% CI 1.18–2.58), compared to US born Black women.

**Table 4:** Association between foreign-born (FB) status and gestational diabetes (GDM), by years in US, stratified by race and ethnicity.

	GDM– N, (%)	GDM+ N (%)	^a^Model 1 RR, (95% CI)	^b^Model 2 RR, (95% CI)	^c^Model 3 RR, (95% CI)
Entire cohort					
US born	15,160 (93.7)	1019 (6.4)	Ref	Ref	Ref
FB with 0–5 years	6348 (91.7)	573 (8.3)	1.32 (1.19–1.46)	1.33 (1.20–1.48)	1.27 (1.14–1.42)
FB with 6–10 years	3358 (86.0)	545 (14.0)	2.22 (2.00–2.60)	2.08 (1.84–2.31)	1.89 (1.68–2.11)
FB with > 10 years	6388 (84.3)	1192 (15.7)	2.50 (2.30–2.71)	2.05 (1.88–2.23)	1.85 (1.69–2.03)
Non-Hispanic Black women					
US born	3624 (94.7)	204 (5.3)	Ref	Ref	Ref
FB with 0–5 years	721 (93.4)	51 (6.6)	1.24 (0.91–1.69)	0.96 (0.71–1.32)	1.10 (0.79–1.55)
FB with 6–10 years	181 (90.1)	20 (10.0)	1.88 (1.19–2.97)	1.37 (0.86–2.18)	1.57 (0.97–2.53)
FB with > 10 years	245 (88.5)	32 (11.6)	2.17 (1.49–3.14)	1.48 (1.01–2.18)	1.75 (1.18–2.58)
Hispanic women					
US born	4989 (92.3)	416 (7.7)	Ref	Ref	Ref
FB with 0–5 years	4869 (91.8)	435 (8.2)	1.07 (0.93–1.22)	0.97 (0.85–1.11)	1.01 (0.87–1.16)
FB with 6–10 years	2828 (85.9)	466 (14.2)	1.84 (1.61–2.10)	1.52 (1.33–1.73)	1.46 (1.27–1.69)
FB with > 10 years	5269 (83.4)	1047 (16.6)	2.16 (1.92–2.41)	1.52 (1.35–1.70)	1.45 (1.28–1.64)
Non-Hispanic Asian women					
US born	465 (91.2)	45 (8.8)	Ref	Ref	Ref
FB with 0–5 years	389 (85.7)	65 (14.3)	1.63 (1.12–2.39)	1.86 (1.27–2.73)	1.49 (0.98–2.26)
FB with 6–10 years	208 (83.9)	40 (16.1)	1.83 (1.23–2.73)	1.86 (1.22–2.85)	1.68 (1.08–2.60)
FB with > 10 years	522 (86.9)	79 (13.1)	1.40 (1.04–2.16)	1.39 (0.96–2.01)	1.37 (0.94–1.99)
Non-Hispanic White women					
US born	5995 (94.5)	349 (5.5)	Ref	Ref	Ref
FB with 0–5 years	262 (95.6)	12 (4.4)	0.81 (0.46–1.45)	0.78 (0.44–1.38)	0.90 (0.50–1.60)
FB with 6–10 years	109 (88.6)	14 (11.4)	2.08 (1.22–3.54)	1.89 (1.11–3.24)	2.27 (1.33–3.88)
FB with > 10 years	298 (91.7)	27 (8.3)	1.50 (1.02–2.22)	1.35 (0.91–2.01)	1.48 (1.00–2.20)

^a^Model 1 was unadjusted/crude.

^b^Model 2 adjusted for maternal age.

^c^Model 3 adjusted for maternal age, chronic health conditions, insurance type, and pre-pregnancy body mass index.

Foreign-born White women with 6–10 years in the US showed an increased risk of GDM (RRadj. 2.27, 95% CI 1.33–3.88), while those with > 10 years had a borderline association with GDM (RRadj. 1.48, 95% CI 1.00–2.20) and those with 0–5 years showed no association. Lastly, foreign-born Asian women with 6–10 years in the US had a higher risk of GDM (RRadj. 1.68, 95% CI 1.08–2.60), but the other groups showed no significant association with GDM compared to US-born Asians.

### Association between acculturation and adverse birth outcomes

The prevalence of other adverse birth outcomes stratified by race/ethnicity can be found in Supplementary Table S1. Among our entire cohort, foreign-born status was associated with a lower risk of adverse birth outcomes, with a few exceptions (Supplementary Table S2). Foreign-born women with 0–5 years in the US had a lower risk of preeclampsia (RRadj. 0.87, 95% CI 0.79–0.96) as well as those at 6–10 years (RRadj. 0.89, 95% CI 0.79–0.99). But those > 10 years in the US showed no association with preeclampsia (RRadj. 1.03, 95% CI 0.95–1.12). Medically indicated preterm birth followed a similar pattern where foreign-born women with 0–5 years (RRadj. 0.62, 95% CI 0.53–0.72) and 6–10 years (RR 0.71, 95% CI 0.59–0.85) had a lower risk but not those with > 10 years in the US. With preeclampsia with severe features, we observed the opposite trend, foreign-born women with 6–10 years (RRadj. 1.42, 95% CI 1.10–1.84) and > 10 years in the US (RRadj. 1.48, 95% CI 1.19–1.83) had an elevated risk, while the 0–5-year group showed no association (RRadj. 1.05, 95% CI 0.83–1.33). Spontaneous preterm birth showed a trend towards higher risk across all acculturative levels in foreign-born women; however, this effect was attenuated in the fully adjusted model. Foreign-born women at all acculturative levels were at lower risk for gestational hypertension, superimposed preeclampsia, and Cesarean section. Results were relatively similar when we stratified by GDM status (Supplementary Table S3).

### Sensitivity analyses

We conducted several sensitivity analyses. First, we examined the association between years in the US as a continuous variable and GDM among foreign-born women. Within the entire cohort, each additional year in the US resulted in a moderate increase in GDM risk (βadj. 0.01, 95% CI 0–0.02, p = 0.01) (Supplementary Table S4). Among foreign-born Hispanic women, each additional year in the US moderately increased GDM risk (βadj. 0.01, 95% CI 0–0.02 p-value 0.01). However, among Black, Asian, and White foreign-born women there was no association.

Next, we conducted a mediation analysis to determine whether prior GDM was a mediator in the association between FBS and GDM. The total causal effect of prior GDM was 2.48, 95% CI 2.20–2.77, and the proportion mediated was 0.53. Based on these results, we concluded that prior GDM was a mediator in the association between FBS and GDM.

## Discussion

We observed that foreign-born women have a higher risk of GDM compared to US-born women across our entire cohort and in all race/ethnic groups. Furthermore, when examining the effect of acculturation on the risk of GDM among foreign-born women, we found a positive relationship between increasing acculturation and GDM risk. Lastly, we observed that foreign-born women across all three acculturative levels were at lower risk for adverse birth outcomes with a few exceptions, mainly preeclampsia with severe features, which was elevated at higher levels of acculturation.

Similar studies conducted within the United States^25^ and other countries, such as Norway^26^ and Denmark^27^, show an elevated risk of GDM among foreign-born women and a positive association between acculturation and GDM risk. Although, Ogunwole et al.^25^ found no significant association between foreign-born women with less than 10 years in the US and GDM. In contrast, we observed foreign-born women with both 0–5 and 6–10 years in US, had elevated risk of GDM compared to US-born women, although risk was highest at > 10 years in the US and we found a modest increase in GDM per yearly increase of residence in the US. One potential theory explaining this effect is the negative acculturation theory^28^, which follows a social conformity framework. This viewpoint hypothesizes that a longer duration in a new country leads to higher exposure to social norms and further integration with the native-born populace. Within the US, this could be seen with the adoption of a more sedentary lifestyle and high physical inactivity levels among immigrants^29^, which has been shown to increase GDM risk^30^. Furthermore, changes in dietary patterns, such as the consumption of sugar-sweetened food and beverages, which are prevalent in the US, could lead to increased risk of weight gain, Type 2 diabetes and pregnancy complications like GDM^31–33^.

There are several factors that could be contributing to our results, showing an increased risk of GDM and preeclampsia with severe features among foreign-born women, particularly at increased acculturative levels. One is the presence of significant barriers to accessing healthcare, along with a lack of familiarity with the system and anti-immigration policies, which has been shown to lead to worse long-term health outcomes^34–36^. Also, the lessening of the protective effect from the healthy migrant effect, along with high levels of stress levels due to high levels of prejudice and discrimination, could contribute to worse birth outcomes for our 6–10 and > 10 year foreign-born groups^37^. However, for other adverse outcomes, we observed a lower risk among foreign-born women. But in the case of preeclampsia this protective effect appeared to wane with increasing acculturation.

Our study has several strengths; over 99% of our population was screened for GDM, in contrast with birth record data, where previous studies have found evidence of underreporting of maternal risk factors and pregnancy complications, like diabetes and preeclampsia^38, 39^. Peribank recruits about 85% of eligible expecting mothers within the Houston area and our foreign-born populace was highly diverse with a large enough sample size to examine various geographical regions or birth origins. Some of our study's limitations include a need for a quantitative measure of the stress and discrimination our foreign-born population experiences. Another limitation is the lack of a standard measure of acculturation, like a validated acculturation scale^40^. However, length of stay is a highly reliable way of quantifying acculturation by proxy^41^. Another limitation is our lack of data on glucose levels within our population. Due to this we were unable to evaluate the association between FBS and glucose levels. We did not have any records indicating when or where prior GDM was diagnosed. We suspect the same criteria was applied among patients diagnosed and treated in the US, however we have a diverse foreign-born population and the diagnostic criteria for GDM varies by country. Lastly, there is potential selection bias because women with uncertain legal status or high levels of mistrust may not consent to be included in the database. This group could be associated with low levels of prenatal care, leading to an underestimation of the effect in our results. Although we excluded women with missing foreign-born status and length of residence data, we did not observe much difference in GDM prevalence between the missing group and our study population (8.9% vs. 9.6%).

In conclusion, we observed a positive relationship between increased acculturation and GDM risk among foreign-born women across multiple race/ethnic groups. Future research could examine the impact of early interventions, specifically within their first 5 years of residence, in lowering the GDM risk among foreign-born women. Future research is also needed to understand social factors that may drive increased risk of GDM among foreign-born women.

## Supplementary Information

Supplementary Tables.

Supplementary Figure S5.

Supplementary Legends.

## Data Availability

The data used in this manuscript was provided to by Peribank under request/license. Access to this data, requires permission from Peribank.

## References

[1] Lawrence RL, Wall CR, Bloomfield FH (2019). Prevalence of gestational diabetes according to commonly used data sources: An observational study. BMC Pregn. Childbirth.

[2] Casagrande SS, Linder B, Cowie CC (2018). Prevalence of gestational diabetes and subsequent Type 2 diabetes among U.S. women. Diabetes Res. Clin. Pract..

[3] Ye W (2022). Gestational diabetes mellitus and adverse pregnancy outcomes: Systematic review and meta-analysis. BMJ.

[4] Pu J (2015). Racial/ethnic differences in gestational diabetes prevalence and contribution of common risk factors. Paediatr. Perinat. Epidemiol..

[5] Razum O, Zeeb H, Rohrmann S (2000). The ‘healthy migrant effect’–not merely a fallacy of inaccurate denominator figures. Int. J. Epidemiol..

[6] Blau FD (2015). Immigrants and gender roles: Assimilation vs. culture. IZA J. Migr..

[7] Chen L, Shi L, Zhang D, Chao SM (2019). Influence of acculturation on risk for gestational diabetes among Asian women. Prevent. Chron. Dis..

[8] Kandula NR (2008). Association of acculturation levels and prevalence of diabetes in the multi-ethnic study of atherosclerosis (MESA). Diabetes Care.

[9] O'Connor MY (2014). Worse cardiometabolic health in African immigrant men than African American Men: Reconsideration of the healthy immigrant effect. Metab. Syndr. Relat. Disord..

[10] Afable-Munsuz A, Mayeda ER, Pérez-Stable EJ, Haan MN (2013). Immigrant generation and diabetes risk among Mexican Americans: The sacramento area latino study on Aging. Am. J. Public Health.

[11] Antony KM (2016). Generation and validation of a universal perinatal database and biospecimen repository: PeriBank. J. Perinatol..

[12] Riosmena F, Kuhn R, Jochem WC (2017). Explaining the immigrant health advantage: Self-selection and protection in health-related factors among five major national-origin immigrant groups in the United States. Demography.

[13] American College of, O. & Gynecologists' Committee on Practice, B.-O. ACOG Practice Bulletin No. 190: Gestational diabetes mellitus.*Obstet. Gynecol.* **131**, e49–e64. 10.1097/AOG.0000000000002501 (2018) 10.1097/AOG.0000000000002501 29370047

[14] Hypertension in pregnancy (2013). Report of the American College of Obstetricians and Gynecologists' Task Force on Hypertension in Pregnancy. Obstet. Gynecol..

[15] American College of, O. & Gynecologists' Committee on Practice, B.-O. Prediction and prevention of spontaneous preterm birth: ACOG Practice Bulletin, Number 234. *Obstet. Gynecol.* **138**, e65–e90. 10.1097/AOG.0000000000004479 (2021) 10.1097/AOG.0000000000004479 34293771

[16] American College of, O. & Gynecologists' Committee on Obstetric Practice, S. f. M.-F. M. Medically indicated late-preterm and early-term deliveries: ACOG Committee Opinion, Number 831. *Obstet. Gynecol.* **138**, e35–e39. 10.1097/AOG.0000000000004447 (2021). 10.1097/AOG.0000000000004447 34259491

[17] Zou G (2004). A modified poisson regression approach to prospective studies with binary data. Am. J. Epidemiol..

[18] McNutt LA, Wu C, Xue X, Hafner JP (2003). Estimating the relative risk in cohort studies and clinical trials of common outcomes. Am. J. Epidemiol..

[19] Textor J, van der Zander B, Gilthorpe MS, Liskiewicz M, Ellison GT (2016). Robust causal inference using directed acyclic graphs: The R package 'dagitty'. Int. J. Epidemiol..

[20] Vanderweele TJ (2019). Principles of confounder selection. Eur. J. Epidemiol..

[21] Textor J, Hardt J, Knuppel S (2011). DAGitty: A graphical tool for analyzing causal diagrams. Epidemiology.

[22] Vanderweele TJ (2016). Mediation analysis: A practitioner's guide. Annu. Rev. Public Health.

[23] Mulla ZD (2007). Spline regression in clinical research. West Indian Med. J..

[24] Billionnet C (2017). Gestational diabetes and adverse perinatal outcomes from 716,152 births in France in 2012. Diabetologia.

[25] Ogunwole SM (2022). Disparities in cardiometabolic risk profiles and gestational diabetes mellitus by nativity and acculturation: Findings from 2016–2017 national health interview survey. BMJ Open Diabetes Res. Care.

[26] Strandberg RB (2021). Gestational diabetes mellitus by maternal country of birth and length of residence in immigrant women in Norway. Diabetic Med..

[27] Kragelund-Nielsen K, Andersen GS, Damm P, Andersen A-MN (2020). Gestational diabetes risk in migrants. A nationwide, register-based study of all births in Denmark 2004 to 2015. J. Clin. Endocrinol. Metabol..

[28] Ro A (2014). The longer you stay, the worse your health? A critical review of the negative acculturation theory among asian immigrants. Int. J. Environ. Res. Public Health.

[29] Singh GK, Yu SM, Siahpush M, Kogan MD (2008). High levels of physical inactivity and sedentary behaviors among US immigrant children and adolescents. Arch. Pediatr. Adolesc. Med..

[30] do-Nascimento GR, Borges MDC, Figueiroa JN, Alves LV, Alves JG (2019). Physical activity pattern in early pregnancy and gestational diabetes mellitus risk among low-income women: A prospective cross-sectional study. SAGE Open Med..

[31] Malik VS, Hu FB (2022). The role of sugar-sweetened beverages in the global epidemics of obesity and chronic diseases. Nat. Rev. Endocrinol..

[32] Casas R, Castro Barquero S, Estruch R (2020). Impact of sugary food consumption on pregnancy: A review. Nutrients.

[33] Luger M (2017). Sugar-sweetened beverages and weight gain in children and adults: A systematic review from 2013 to 2015 and a comparison with previous studies. Obes. Facts.

[34] Page-Reeves J, Regino L, Schleder T (2022). Policy implications of structural violence and syndemic dynamics: A lens for addressing latinx immigrant diabetes health disparities. Curr. Diab. Rep..

[35] Callaghan T (2019). Immigrant health access in Texas: Policy, rhetoric, and fear in the Trump era. BMC Health Serv. Res..

[36] Fuentes-Afflick E, Odouli R, Escobar GJ, Stewart AL, Hessol NA (2014). Maternal acculturation and the prenatal care experience. J. Womens Health.

[37] Elshahat S, Moffat T, Newbold KB (2022). Understanding the healthy immigrant effect in the context of mental health challenges: A systematic critical review. J. Immigr. Minor. Health.

[38] Digiuseppe DL, Aron DC, Ranbom L, Harper DL, Rosenthal GE (2002). Reliability of birth certificate data: A multi-hospitalcomparison to medical records information. Matern. Child Health J..

[39] Northam S, Knapp TR (2006). The reliability and validity of birth certificates. JOGNN.

[40] (US), C. f. S. A. T. *Appendix B, Instruments to Measure Identity and Acculturation*, https://www.ncbi.nlm.nih.gov/books/NBK248425/ (2014).

[41] Wallace PM, Pomery EA, Latimer AE, Martinez JL, Salovey P (2010). A review of acculturation measures and their utility in studies promoting latino health. Hisp. J. Behav. Sci..

