# Supplementary material for: Foreign-born status and risk of gestational diabetes mellitus by years of residence in the United States
Source: Sci Rep. 2023 Jun 21;13:10060. doi: 10.1038/s41598-023-36789-8 (PMC10285025; doi:10.1038/s41598-023-36789-8)
Supplement: Supplementary file 1 — Supplementary Tables. [file 41598_2023_36789_MOESM1_ESM.docx]

| **Supplemental Table S1. Prevalence of adverse birth outcomes, stratified by race and ethnicity** | | | | | |
| --- | --- | --- | --- | --- | --- |
| **Outcomes** | **Entire Cohort**  **N (%)** | **Non-Hispanic Black Women**  **N (%)** | **Hispanic Women**  **N (%)** | **Non-Hispanic Asian Women**  **N (%)** | **Non-Hispanic White Women**  **N (%)** |
| Preeclampsia | 4071 (11.7) | 727 (14.3) | 1448 (12.0) | 114 (6.3) | 764 (10.8) |
| Preeclampsia with severe features | 633 (1.8) | 102 (2.0) | 448(2.2) | 15 (0.8) | 65 (0.9) |
| Superimposed preeclampsia | 1393 (4.0) | 287 (5.6) | 691 (3.4) | 43 (2.4) | 366 (5.2) |
| Gestational hypertension | 3042 (8.7) | 574 (11.3) | 1702(8.4) | 96 (5.3) | 657 (9.3) |
| Cesarean section | 11557 (33.3) | 2185 (42.9) | 6013 (29.5) | 648 (35.7) | 2594 (36.6) |
| Spontaneous preterm birth | 5110 (3.0) | 770 (3.0) | 3530 (3.5) | 170 (1.9) | 580 (1.6) |
| Medically indicated preterm birth | 1699 (4.9) | 311 (6.1) | 953 (4.7) | 84 (4.6) | 349 (4.9) |

| **Supplemental Table 2. Association between foreign-born (FB) status and adverse birth outcomes, by years in US: results from the entire cohort** | | | | |
| --- | --- | --- | --- | --- |
| **Outcomes** | **FB Status** | **^a^Model 1**  **RR (95% CI)** | **^b^Model 2**  **RR (95% CI)** | **^c^Model 3**  **RR (95% CI)** |
| Preeclampsia | US Born | Ref. | Ref. | Ref. |
|  | FB with 0 – 5 years | 0.79 (0.73 – 0.86) | 0.79 (0.73 – 0.86) | 0.87 (0.79 – 0.96) |
|  | FB with 6 – 10 years | 0.86 (0.78 – 0.95) | 0.85 (0.77 – 0.95) | 0.89 (0.79 – 0.99) |
|  | FB with >10 years | 1.04 (0.97 – 1.12) | 1.02 (0.94 – 1.10) | 1.03 (0.95 – 1.12) |
|  |  |  |  |  |
| Preeclampsia with severe features | US Born | Ref. | Ref. | Ref. |
|  | FB with 0 – 5 years | 1.27 (1.12 – 1.02) | 1.26 (1.01 – 1.58) | 1.05 (0.83 – 1.33) |
|  | FB with 6 – 10 years | 1.80 (1.42 – 2.28) | 1.81 (1.43 – 2.30) | 1.42 (1.10 – 1.84) |
|  | FB with >10 years | 1.84 (1.52 – 2.22) | 1.87 (1.53 – 2.27) | 1.48 (1.19 – 1.83) |
|  |  |  |  |  |
| Superimposed preeclampsia | US Born | Ref. | Ref. | Ref. |
|  | FB with 0 – 5 years | 0.43 (0.36 – 0.51) | 0.44 (0.37 – 0.52) | 0.54 (0.45 – 0.65) |
|  | FB with 6 – 10 years | 0.58 (0.48 – 0.70) | 0.56 (0.46 - 0.69) | 0.67 (0.54 – 0.82) |
|  | FB with >10 years | 0.76 (0.66 – 0.87) | 0.70 (0.61 – 0.80) | 0.80 (0.69 – 0.92) |
|  |  |  |  |  |
| Gestational hypertension | US Born | Ref. | Ref. | Ref. |
|  | FB with 0 – 5 years | 0.71 (0.65 – 0.79) | 0.72 (0.65 – 0.79) | 0.76 (0.68 - 0.85) |
|  | FB with 6 – 10 years | 0.80 (0.71 – 0.90) | 0.80 (0.71 – 0.90) | 0.82 (0.72 – 0.93) |
|  | FB with >10 years | 0.90 (0.82 – 0.98) | 0.88 (0.80 – 0.97) | 0.89 (0.80 – 0.98) |
|  |  |  |  |  |
| Cesarean section | US Born | Ref. | Ref. | Ref. |
|  | FB with 0 – 5 years | 0.82 (0.79 – 0.86) | 0.83 (0.79 – 0.88) | 0.90 (0.85 – 0.95) |
|  | FB with 6 – 10 years | 0.78 (0.74 – 0.82) | 0.75 (0.70 – 0.80) | 0.80 (0.74 – 0.85) |
|  | FB with >10 years | 0.81 (0.78 – 0.84) | 0.73 (0.69 – 0.76) | 0.76 (0.72 – 0.80) |
|  |  |  |  |  |
| Spontaneous Preterm Birth | US Born | Ref. | Ref. | Ref. |
|  | FB with 0 – 5 years | 1.34 (1.14 – 1.58) | 1.34 (1.14 – 1.57) | 1.06 (0.89 – 1.26) |
|  | FB with 6 – 10 years | 1.36 (1.12 – 1.66) | 1.38 (1.13 – 1.68) | 1.07 (0.87 – 1.32) |
|  | FB with >10 years | 1.41 (1.21 – 1.65) | 1.46 (1.24 – 1.71) | 1.16 (0.98 – 1.38) |
|  |  |  |  |  |
| Medically indicated Preterm Birth | US Born | Ref. | Ref. | Ref. |
|  | FB with 0 – 5 years | 0.59 (0.51 – 0.68) | 0.60 (0.51 – 0.69) | 0.62 (0.53 – 0.72) |
|  | FB with 6 – 10 years | 0.73 (0.62 – 0.86) | 0.70 (0.59 – 0.83) | 0.71 (0.59 – 0.85) |
|  | FB with >10 years | 1.01 (0.90 – 1.13) | 0.90 (0.80 – 1.01) | 0.88 (0.78 – 1.01) |
| ^a^Model 1 was unadjusted/crude  ^b^Model 2 adjusted for maternal age  ^c^Model 3 adjusted for maternal age, chronic health conditions, insurance type, and pre-pregnancy body mass index | | | | |

| **Supplemental Table 3.** **Association between foreign-born (FB) status and adverse birth outcomes, by years in US, stratified by gestational diabetes (GDM)** | | | | | | | |
| --- | --- | --- | --- | --- | --- | --- | --- |
|  |  | **GDM +**  **N,% = 3329, 9.6%** | | | **GDM –**  **N, % = 31254, 90.4%** | | |
| **Outcomes** | **FB Status** | **^a^Model 1**  **RR (95% CI)** | **^b^Model 2**  **RR (95% CI)** | **^c^Model 3**  **RR (95% CI)** | **^a^Model 1**  **RR (95% CI)** | **^b^Model 2**  **RR (95% CI)** | **^c^Model 3**  **RR (95% CI)** |
| Preeclampsia | US Born | Ref | Ref | Ref | Ref | Ref | Ref |
|  | FB with 0 – 5 years | 1.03 (0.81 – 1.31) | 1.03 (0.81 – 1.31) | 1.02 (0.78 – 1.34) | 0.75 (0.68 – 0.82) | 0.75 (0.68 – 0.83) | 0.84 (0.76 – 0.94) |
|  | FB with 6 – 10 years | 0.96 (0.74 – 1.23) | 0.95 (0.73 – 1.22) | 0.90 (0.68 – 1.18) | 0.80 (0.71 – 0.90) | 0.80 (0.71 – 0.90) | 0.86 (0.76 – 0.98) |
|  | FB with >10 years | 1.21 (1.00 – 1.47) | 1.19 (0.98 – 1.45) | 1.11 (0.89 – 1.39) | 0.95 (0.87 – 1.03) | 0.93 (0.86 - 1.02) | 0.98 (0.89 - 1.08) |
|  |  |  |  |  |  |  |  |
| Preeclampsia with severe features | US Born | Ref | Ref | Ref | Ref | Ref | Ref |
|  | FB with 0 – 5 years | 1.27 (0.67 – 2.41) | 1.27 (0.67 – 2.41) | 1.17 (0.59 – 2.23) | 1.25 (0.99 – 1.58) | 1.24 (0.98 – 1.57) | 1.03 (0.80 – 1.33) |
|  | FB with 6 – 10 years | 1.84 (1.02 – 3.32) | 1.87 (1.03 – 3.38) | 1.62 (0.85 – 3.09) | 1.69 (1.29 – 2.22) | 1.70 (1.31 – 2.22) | 1.35 (1.02 – 1.79) |
|  | FB with >10 years | 2.06 (1.26 – 3.38) | 2.14 (1.28 – 3.56) | 1.86 (1.06 – 3.27) | 1.63 (1.31 – 2.03) | 1.68 (1.35 – 2.10) | 1.35 (1.07 – 1.71) |
|  |  |  |  |  |  |  |  |
| Superimposed preeclampsia | US Born | Ref | Ref | Ref | Ref | Ref | Ref |
|  | FB with 0 – 5 years | 0.63 (0.41 – 0.97) | 0.63 (0.41 – 0.97) | 0.76 (0.47 – 1.21) | 0.40 (0.33 – 0.48) | 0.40 (0.33 – 0.49) | 0.51 (0.42 – 0.62) |
|  | FB with 6 – 10 years | 0.57 (0.36 – 0.90) | 0.55 (0.35 – 0.86) | 0.60 (0.37 – 0.99**)** | 0.55 (0.45 – 0.69) | 0.55 (0.44 – 0.68) | 0.66 (0.53 – 0.83) |
|  | FB with >10 years | 0.99 (0.73 – 1.34) | 0.89 (0.65 – 1.22) | 0.96 (0.67 – 1.36) | 0.65 (0.55 – 0.76) | 0.61 (0.52 – 0.72) | 0.72 (0.60 – 0.85) |
|  |  |  |  |  |  |  |  |
| Gestational hypertension | US Born | Ref | Ref | Ref | Ref | Ref | Ref |
|  | FB with 0 – 5 years | 0.74 (0.55 – 0.99) | 0.74 (0.55 -0.99) | 0.68 (0.50 – 0.94) | 0.70 (0.63 – 0.78) | 0.70 (0.63 – 0.78) | 0.77 (0.68 – 0.86) |
|  | FB with 6 – 10 years | 0.76 (0.57 – 1.03) | 0.76 (0.57 – 1.02) | 0.67 (0.49 – 0.92) | 0.77 (0.67 – 0.88) | 0.77 (0.67 – 0.88) | 0.82 (0.71 – 0.94) |
|  | FB with >10 years | 0.93 (0.75 – 1.16) | 0.92 (0.73 – 1.16) | 0.80 (0.62 – 1.04) | 0.83 (0.75 – 0.92) | 0.83 (0.74 – 0.91) | 0.86 (0.77 – 0.96) |
|  |  |  |  |  |  |  |  |
| Cesarean section | US Born | Ref | Ref | Ref | Ref | Ref | Ref |
|  | FB with 0 – 5 years | 0.87 (0.75 – 1.02) | 0.88 (0.75 – 1.02) | 0.99 (0.83 – 1.18) | 0.81 (0.77 – 0.86) | 0.83 (0.78 – 0.87) | 0.89 (0.84 – 0.94) |
|  | FB with 6 – 10 years | 0.75 (0.64 - 0.89) | 0.73 (0.61 – 0.86) | 0.81 (0.67 – 0.97) | 0.76 (0.71 – 0.82) | 0.74 (0.69 – 0.80) | 0.79 (0.73 – 0.85) |
|  | FB with >10 years | 0.78 (0.69 – 0.89) | 0.71 (0.62 – 0.82) | 0.79 (0.67 – 0.91) | 0.79 (0.75 – 0.83) | 0.72 (0.68 – 0.75) | 0.75 (0.71 – 0.79) |
|  |  |  |  |  |  |  |  |
| Spontaneous preterm birth | US Born | Ref | Ref | Ref | Ref | Ref | Ref |
|  | FB with 0 – 5 years | 0.98 (0.57 – 1.69) | 0.98 (0.57 – 1.69) | 0.83 (0.46 – 1.50) | 1.38 (1.16 – 1.63) | 1.37 (1.15 – 1.62) | 1.08 (0.90 – 1.30) |
|  | FB with 6 – 10 years | 1.62 (1.00 – 2.62) | 1.59 (0.98 – 2.58) | 1.33 (0.78 – 2.27) | 1.24 (0.99 – 1.55) | 1.26 (1.01 – 1.58) | 0.99 (0.78 – 1.25) |
|  | FB with >10 years | 1.44 (0.95 – 2.17) | 1.37 (0.90 – 2.09) | 1.16 (0.72 – 1.88) | 1.33 (1.12 – 1.58) | 1.39 (1.17 – 1.66) | 1.12 (0.93 – 1.35) |
|  |  |  |  |  |  |  |  |
| Medically indicated preterm birth | US Born | Ref | Ref | Ref | Ref | Ref | Ref |
|  | FB with 0 – 5 years | 0.56 (0.37 – 0.83) | 0.56 (0.37 – 0.83) | 0.66 (0.43 – 1.02) | 0.58 (0.50 – 0.68) | 0.59 (0.51 – 0.69_ | 0.60 (0.51 – 0.71) |
|  | FB with 6 – 10 years | 0.79 (0.55 – 1.13) | 0.76 (0.53 – 1.10) | 0.87 (0.59 – 1.30) | 0.65 (0.54 – 0.79) | 0.64 (0.52 – 0.77) | 0.64 (0.52 – 0.78) |
|  | FB with >10 years | 0.90 (0.68 – 1.19) | 0.83 (0.62 – 1.10) | 0.92 (0.66 – 1.27) | 0.95 (0.83 – 1.08) | 0.86 (0.76 – 0.98) | 0.85 (0.73 – 0.98) |
| ^a^Model 1 was unadjusted/crude  ^b^Model 2 adjusted for maternal age  ^c^Model 3 adjusted for maternal age, chronic health conditions, insurance type, and pre-pregnancy body mass index | | | | | | | |

| **Supplemental Table 4. Association between years in US (continuous) and gestational diabetes among foreign-born (FB) women, stratified by race and ethnicity** | | | |
| --- | --- | --- | --- |
|  | **^a^Model 1**  **β (95% CI)**  ***P*** | **^b^Model 2**  **β (95% CI)**  ***P*** | **^c^Model 3**  **β (95% CI)**  ***P*** |
| Entire Cohort | 0.02 (0.02 – 0.03)  <0.001 | 0.01 (0.01 – 0.02)  <0.001 | 0.01 (0.00 – 0.02)  0.01 |
| Non-Hispanic Black FB women | 0.02 (0.00 – 0.04)  0.06 | 0.02 (-0.01 – 0.04)  0.17 | 0.02 (-0.01 – 0.06)  0.13 |
| Hispanic FB women | 0.03 (0.03 – 0.04)  <0.001 | 0.02 (0.01 – 0.02)  <0.001 | 0.01 (0.00 – 0.02)  0.003 |
| Non-Hispanic Asian FB women | -0.01 (-0.02 – 0.01)  0.28 | -0.02 (-0.03 – (-0.01))  0.04 | -0.01 (-0.02 – 0.01)  0.38 |
| Non-Hispanic White FB women | 0.01 (-0.02 – 0.03)  0.52 | 0.00 (-0.02 – 0.03)  0.72 | 0.01 (-0.02 - (-0.03))  0.58 |
| ^a^Model 1 was unadjusted  ^b^Model 2 adjusted for maternal age  ^c^Model 3 adjusted for maternal age, chronic health conditions, insurance type, and pre-pregnancy body mass index | | | |
