# Supplementary figures and images for: Foreign-born status and risk of gestational diabetes mellitus by years of residence in the United States
Source: Sci Rep. 2023 Jun 21;13:10060. doi: 10.1038/s41598-023-36789-8 (PMC10285025; doi:10.1038/s41598-023-36789-8)

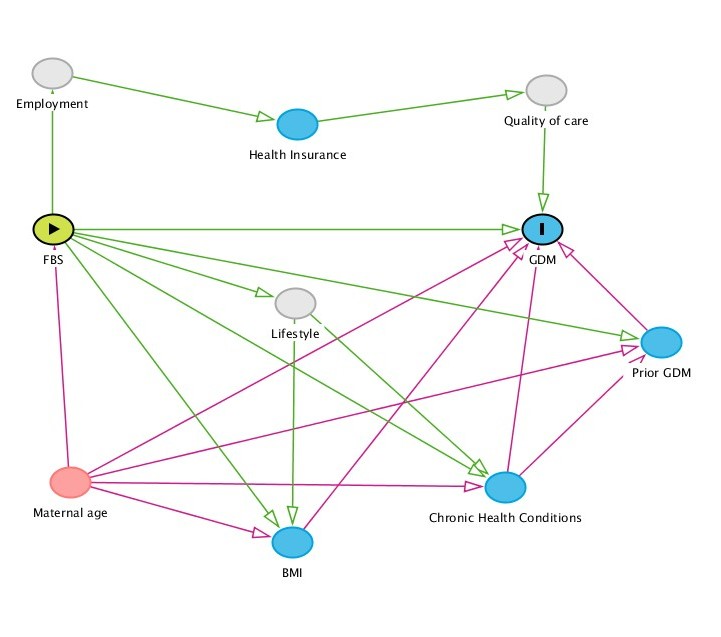

Supplement: Supplementary file 2 — Supplementary Figure S5. [file 41598_2023_36789_MOESM2_ESM.jpg]
