# Supplementary material for: Foreign-born status and risk of gestational diabetes mellitus by years of residence in the United States
Source: Sci Rep. 2023 Jun 21;13:10060. doi: 10.1038/s41598-023-36789-8 (PMC10285025; doi:10.1038/s41598-023-36789-8)
Supplement: Supplementary file 3 — Supplementary Legends. [file 41598_2023_36789_MOESM3_ESM.docx]

**Supplementary Figure S5**. Directed acyclic graph of the association between foreign-born status (FBS) and gestational diabetes (GDM).
